# Associations of *PRKN–PACRG* SNPs and G × G and G × E interactions with the risk of hyperlipidaemia

**DOI:** 10.1038/s41598-020-68826-1

**Published:** 2020-08-03

**Authors:** Peng-Fei Zheng, Rui-Xing Yin, Bi-Liu Wei, Chun-Xiao Liu, Guo-Xiong Deng, Yao-Zong Guan

**Affiliations:** 1 Department of Cardiology, Institute of Cardiovascular Diseases, The First Affiliated Hospital, Guangxi Medical University, Nanning, 530021 Guangxi People’s Republic of China; 2 Guangxi Key Laboratory Base of Precision Medicine in Cardio-Cerebrovascular Disease Control and Prevention, Nanning, 530021 Guangxi People’s Republic of China; 3 Guangxi Clinical Research Center for Cardio-Cerebrovascular Diseases, Nanning, 530021 Guangxi People’s Republic of China

**Keywords:** Genetics, Genetic association study

## Abstract

This research aimed to assess the associations of 7 parkin RBR E3 ubiquitin protein ligase (*PRKN*) and 4 parkin coregulated gene (*PACRG*) single-nucleotide polymorphisms (SNPs), their haplotypes, gene–gene (G × G) and gene-environment (G × E) interactions with hyperlipidaemia in the Chinese Maonan minority. The genotypes of the 11 SNPs in 912 normal and 736 hyperlipidaemic subjects were detected with next-generation sequencing technology. The genotypic and allelic frequencies of the rs1105056, rs10755582, rs2155510, rs9365344, rs11966842, rs6904305 and rs11966948 SNPs were different between the normal and hyperlipidaemic groups (*P* < 0.05–0.001). Correlations between the above 7 SNPs and blood lipid levels were also observed (*P* < 0.0045–0.001, *P* < 0.0045 was considered statistically significant after Bonferroni correction). Strong linkage disequilibrium was found among the 11 SNPs (*r*^2^ = 0.01–0.64). The most common haplotypes were *PRKN* C-G-T-G-T-T-C (> 15%) and *PACRG* A-T-A-T (> 40%). The *PRKN* C-G-C-A-T-T-C and *PRKN–PACRG* C-G-T-G-T-T-C-A-T-A-T haplotypes were associated with an increased risk of hyperlipidaemia, whereas the *PRKN–PACRG* C-G-T-G-C-T-C-A-T-C-T and C-G-T-G-T-T-C-A-T-C-T haplotypes provided a protective effect. Association analysis based on the haplotypes and G × G interaction could improve the power to detect the risk of hyperlipidaemia over the analysis of any one SNP alone. The differences in serum lipid parameters between the hyperlipidaemic and normal groups might partly be due to the effects of the *PRKN–PACRG* SNPs and their haplotypes.

## Introduction

Coronary artery disease (CAD) has become a prominent cause of morbidity, mortality, disability, high healthcare costs and functional deterioration and accounts for approximately 30% of all deaths worldwide^1–3^. Hyperlipidaemia is a major risk factor for CAD and its complications. Comprehensive lipid-lowering therapy is recommended for patients with CAD by the 2013 American College of Cardiology (ACC)/American Heart Association (AHA) guidelines for the treatment of blood cholesterol to reduce the risk of cardiovascular events^4^. The guidelines emphasize that lipid-lowering therapy should not focus solely on decreasing low-density lipoprotein cholesterol (LDL-C) levels. Several compelling studies proved that lowering total cholesterol (TC)^5^, triglyceride (TG)^5^ and LDL-C^6^ levels is more effective in reducing cardiovascular risk than lowering LDL-C levels alone^7^. The “6 percent effect” of statins refers to the fact that doubling the dose of statins only decreases LDL-C levels by 6.4%, and PCSK9 inhibitors combined with statins are recommended for acute coronary syndrome (ACS) patients with a high risk of cardiovascular events^8^. Hyperlipidaemia is a highly hereditary disease, and 40–60% of the variation in blood lipid spectra is genetically determined^9,10^; hence, it is important to identify novel lipid-related genes to guide the development of new lipid-lowering drugs. Recently, several compelling genes that are closely associated with blood lipid levels, including the parkin RBR E3 ubiquitin protein ligase gene (*PRKN* [MIM602544]) and the parkin coregulated gene (*PACRG* [MIM608427]), have been identified by genome-wide association studies (GWASes) in the Hutterites, a founder population of European descent^11^. Previous studies indicated that *PRKN* mutations associated with mitochondrial dysfunction are implicated in the development of metabolic syndrome, including hypercholesterolemia, hypertension, steatohepatitis, obesity and glucose intolerance^12,13^. *PACRG* is linked with the adjacent *PRKN* gene in a head-to-head arrangement, so these two genes may have similar biological functions. Li et al*.*^14^ reported that the *PRKN* and *PACRG* genes are associated with susceptibility to leprosy. According to Silva et al*.* ^15^, lipid metabolism plays a crucial role in the pathological process of leprosy, and lipids are related to a higher risk of cardiovascular events in subjects suffering from leprosy.

China is a country with multiple ethnicities, including the Han nationality and 55 ethnic minorities. The sixth national census statistics of China (2010) showed that the total population of the Maonan ethnic group was 107,166 (37th). Most of the Maonan population is located in Huanjiang Maonan Autonomous County, Guangxi Zhuang Autonomous Region. Although Maonan and Han people live in the same area, there are various differences in lifestyle and dietary habits between the Maonan and local Han populations^16^. A previous study showed that the rs9534275 SNP of *BRCA2* is related to serum apolipoprotein (Apo) B, TC, and LDL-C levels in the Maonan population with hyperlipidaemia^17^, but associations between other genetic polymorphisms and hyperlipidaemia have not been found in this ethnic group. Thus, this study was designed to determine the association of *PRKN–PACRG* SNPs, their haplotypes, and gene–gene (G × G), haplotype-haplotype, gene-environment (G × E), and haplotype-environment interactions with serum lipid levels in the Chinese Maonan ethnic group, a relatively conservative and isolated minority.

## Results

### Common and biochemical characteristics

As mentioned in Table 1, the levels of ApoB, TG, LDL-C, TC, systolic blood pressure and the proportion of smokers, diastolic blood pressure, pulse pressure, and blood glucose were greater in hyperlipidemic than in normal groups (*P* < 0.05–0.001). The levels of serum ApoA1, high-density lipoprotein cholesterol (HDL-C), height and the ApoA1/ApoB ratio were less in hyperlipidemic than in normal groups (*P* < 0.05–0.001). There was no any obvious difference in the factors including age distribution, gender ratio, waist circumference, weight, body mass index (BMI), alcohol consumption between the hyperlipidemic and normal groups.

**Table 1:** Comparison of demographic, lifestyle characteristics and serum lipid levels between the normal and hyperlipidemic populations.

Parameter	Normal	Hyperlipidemia	*t* (*x*^2^)	*P*
Number	912	736		
Male/female	405/507	330/406	0.030	0.862
Age (years)	55.83 ± 15.48	55.55 ± 14.33	− 0.375	0.708
Height (cm)	155.85 ± 8.66	154.85 ± 10.61	− 2.121	0.034
Weight (kg)	55.40 ± 12.30	56.43 ± 13.40	1.627	0.104
Body mass index (kg/m^2^)	22.71 ± 4.27	23.59 ± 4.42	1.316	0.205
Waist circumference	76.36 ± 10.05	76.87 ± 9.67	1.050	0.294
**Smoking status [n (%)]**				
Non-smoker	730 (80.04)	560 (76.09)		
≤ 20 cigarettes/day	81 (8.88)	136 (18.48)		
> 20 cigarettes/day	101 (11.07)	40 (5.43)	44.444	2.23E−10
**Alcohol consumption [n (%)]**				
Non-drinker	734 (77.57)	580 (79.15)		
≤ 25 g/day	66 (11.57)	63 (11.18)		
> 25 g/day	112 (10.86)	93 (9.68)	1.096	0.578
Systolic blood pressure (mmHg)	129.58 ± 22.07	137.27 ± 20.86	7.041	2.79E−12
Diastolic blood pressure (mmHg)	79.98 ± 12.60	84.48 ± 12.00	7.362	2.85E−13
Pulse pressure (mmHg)	49.63 ± 16.43	52.80 ± 16.70	3.856	1.19E−4
Glucose (mmol/L)	6.01 ± 1.16	6.24 ± 1.35	3.621	3.03E−4
Total cholesterol (mmol/L)	4.26 ± 0.64	5.68 ± 1.21	32.056	1.05E−162
Triglyceride (mmol/L)	1.06 (0.45)	1.93 (1.31)	− 24.107	8.21E−14
HDL-C (mmol/L)	1.56 ± 0.49	1.46 ± 0.46	− 3.826	1.35E−4
LDL-C (mmol/L)	2.49 ± 0.54	3.35 ± 0.79	18.318	5.78E−66
ApoA1 (g/L)	1.37 ± 0.22	1.32 ± 0.26	− 3.671	2.50E−4
ApoB (g/L)	0.80 ± 0.16	1.0 ± 0.19	23.861	4.35E−103
ApoA1/ApoB	1.76 ± 0.44	1.39 ± 0.51	− 15.57	4.25E−51

*HDL-C* high-density lipoprotein cholesterol, *LDL-C* low-density lipoprotein cholesterol, *Apo* apolipoprotein.

^a^Mean ± SD determined by *t* test.

^b^The value of triglyceride was presented as median (interquartile range), the difference between the two groups was determined by the Wilcoxon–Mann–Whitney test.

### Genotypic and allelic occurrence of SNPs and the association with serum lipid levels

The genotypic and allelic occurrences of the SNPs within *PRKN* (rs10755582, rs9458363, rs2022991, rs9365344, rs1105056, rs4636000 and rs2155510) and *PACRG* (rs2206256, rs11966842, rs11966948 and rs6904305) are represented in Table 2. The genotype distribution of all 11 SNPs was consistent with Hardy–Weinberg equilibrium (HWE) in both hyperlipidemic and normal groups (*P* > 0.05 for all). The genotypic and allelic frequencies of 7 SNPs (*PRKN* rs10755582, rs9365344, rs1105056, rs2155510 and *PACRG* rs11966842, rs11966948 and rs6904305) were significantly different between the two groups (*P* < 0.05–0.001).

**Table 2:** The association between the *PRKN*, *PACRG* polymorphisms with hyperlipidemia [n (%)].

SNP	Genotype	Normal (*n* = 912)	Hyperlipidemia (*n* = 736)	*x*^2^	*P*_1_	OR (95% CI)	*P*_2_
*PRKN*	TT	271 (29.7)	181 (24.6)			1	–
rs1105056 C > T	CT + CC	641 (70.3)	555 (75.4)	5.369	0.020	1.24 (0.97–1.57)	0.032
	MAF	839 (46.0)	721 (49.0)	2.908	0.088		
	*P*_HWE_	0.51	0.14				
*PRKN*	TT	438 (48.0)	355 (48.2)			1	
rs4636000 C > T	CT + CC	474 (52.0)	381 (51.8)	0.007	0.933	0.99 (0.82–1.20)	0.200
	MAF	567 (31.0)	435 (30.0)	0.906	0.341		
	*P*_HWE_	0.44	0.08				
*PRKN*	CC	632 (69.3)	558 (75.8)			1	
rs10755582 C > T	CT + TT	280 (30.7)	178 (24.2)	8.620	0.003	0.59 (0.46–0.76)	2E-04
	MAF	310 (17.0)	196 (13.0)	8.491	0.004		
	*P*_HWE_	0.41	0.11				
*PRKN*	CC	472 (51.8)	300 (40.8)			1	
rs2155510 T > C	TC + TT	440 (48.2)	436 (59.2)	19.769	8.7E-6	1.49 (1.20–1.86)	3E-04
	MAF	513 (28.0)	514 (35.0)	17.527	1.8E-4		
	*P*_HWE_	0.87	0.06				
*PRKN*	GG	367 (40.2)	236 (32.1)			1	
rs9365344 A > G	AG + AA	545 (59.8)	500 (67.9)	11.735	0.001	1.38 (1.10–1.72)	0.005
	MAF	683 (37.0)	620 (42.0)	7.446	0.006		
	*P*_HWE_	0.16	0.13				
*PRKN*	GG	350 (38.4)	272 (37.0)			1	
rs9458363 G > T	GT + TT	562 (61.6)	464 (63.0)	0.350	0.554	1.06 (0.87–1.30)	0.240
	MAF	698 (38.0)	564 (38.0)	0.001	0.978		
	*P*_HWE_	0.73	0.24				
*PRKN*	TT	268 (29.4)	234 (31.8)			1	
rs2022991 C > T	CT + CC	644 (70.6)	502 (68.2)	1.114	0.291	0.85 (0.67–1.07)	0.170
	MAF	821(45.0)	638 (43.0)	0.919	0.338		
	*P*_HWE_	0.32	0.76				
*PACRG*	TT	510 (55.9)	450 (61.1)			1	
rs11966842 C > T	CT + CC	402 (44.1)	286 (38.9)	3.576	0.033	0.71 (0.57–0.88)	0.002
	MAF	459 (25.0)	322 (22.0)	4.875	0.027		
	*P*_HWE_	0.72	0.91				
*PACRG*	TT	552 (60.5)	408 (55.4)			1	
rs6904305 T > G	TG + GG	360 (39.5)	328 (44.6)	4.342	0.037	1.23 (1.01–1.50)	0.017
	MAF	400 (22.0)	368 (25.0)	4.296	0.038		
	*P*_HWE_	0.5	0.28				
*PACRG*	AA	531 (58.2)	448 (60.9)			1	
rs2206256 T > A	TA + TT	381 (41.8)	288 (39.1)	1.182	0.277	0.90 (0.73–1.09)	0.280
	MAF	426 (23.0)	316 (21.0)	1.664	0.197		
	*P*_HWE_						
*PACRG*	AA	289 (31.7)	286 (38.9)			1	
rs11966948 C > A	CA + CC	623 (68.3)	450 (61.1)	9.217	0.002	0.65 (0.52–0.82)	2E-04
	MAF	776 (43.0)	558 (38.0)	7.268	0.007		
	*P*_HWE_	0.12	0.75				

*PRKN* parkin RBR E3 ubiquitin protein ligase gene, *PACRG* the parkin coregulated gene, *HLP* hyperlipidemia, *HWE* Hardy–Weinberg equilibrium. *MAF* minor allele frequency. *P*_1_ was obtained by Chi-square test probability. *P*_2_ was obtained by unconditional logistic regression analysis.

**Figure 1:**
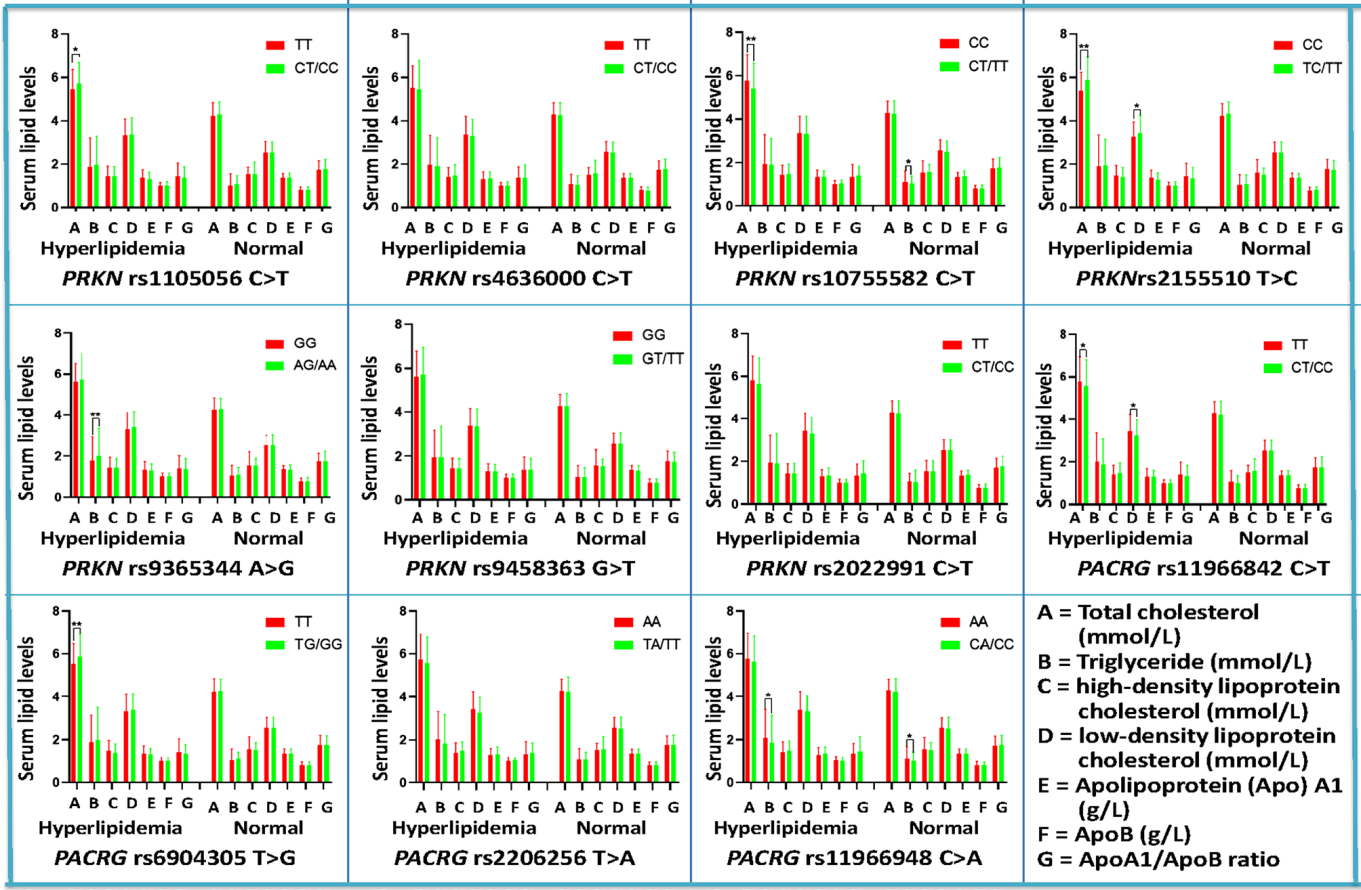
Association between the genotypes of *PRKN* and *PACRG* SNPs and blood lipid levels in the control, HCH and HTG groups. *TC*, Total cholesterol; *TG*, Triglyceride; *HDL-C*, High-density lipoprotein cholesterol; *LDL-C*, Low-density lipoprotein cholesterol; *Apo*, Apolipoprotein. ^*^P < 0.0045; ^**^P < 0.001. (*P* < 0.0045 was considered statistically significant after adjusting by Bonferroni correction).

The dominant models of the rs1105056, rs2155510, rs9365344 and rs6904305 SNPs were associated with an increased morbidity, whereas the dominant models of the rs10755582, rs11966842 and rs11966948 SNPs provided a protective effect (*P* < 0.05–0.001). As shown in Fig. 1, the correlation between the *PRKN* and *PACRG* SNPs and serum lipid parameters including LDL-C (rs11966842 and rs2155510), TC (rs1105056, rs10755582, rs2155510, rs11966842 and rs6904305), and TG (rs9365344 and rs11966948) in subjects with hyperlipidaemia; and TG (rs10755582 and rs11966948) in normal group was observed (*P* < 0.0045–0.001, *P* < 0.0045 was considered statistically significant after Bonferroni correction).

### Haplotype-based association with hyperlipidaemia

**Figure 2:**
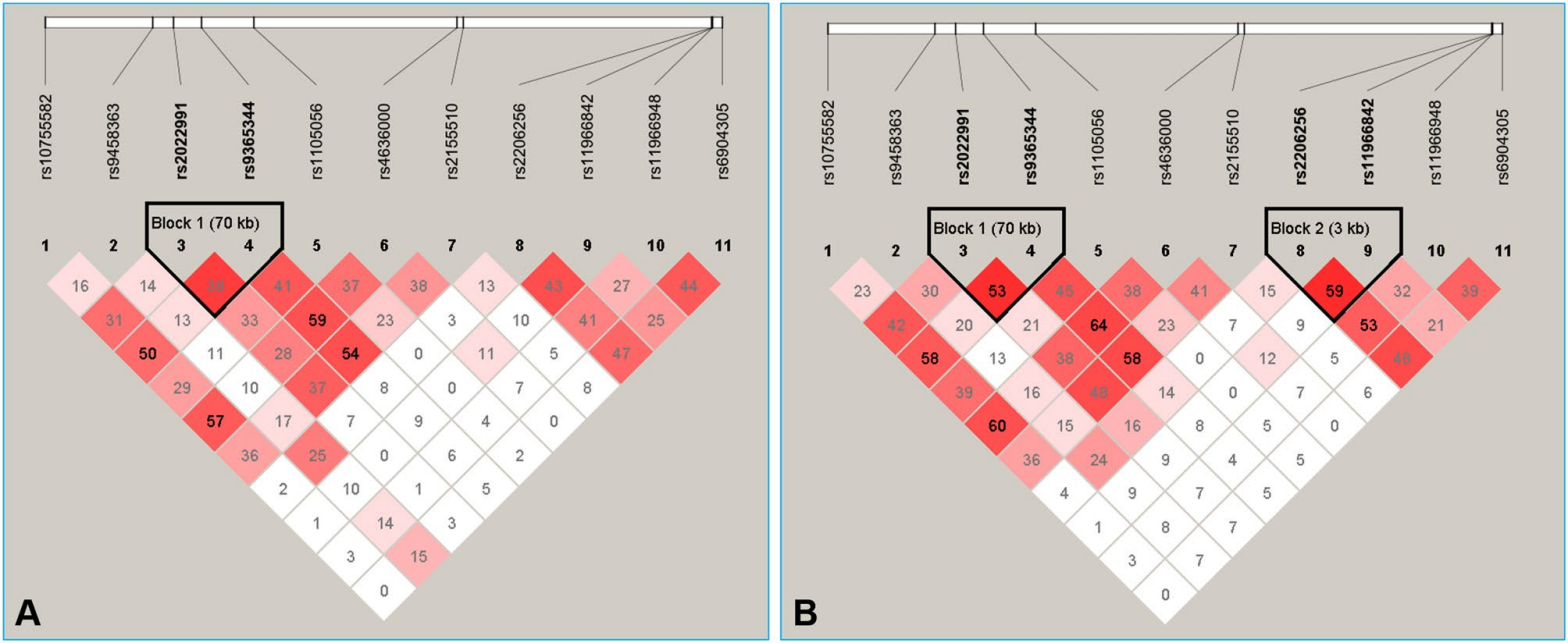
The linkage disequilibrium (LD) represents pair-wise *r*^2^ × 100 in the normal (**A**) and hyperlipidemia (**B**) groups.

As shown in Fig. 2, there was strong pairwise linkage disequilibrium (LD) among the detected loci in Han and Maonan groups. The dominant haplotypes were *PRKN* C-G-T-G-T-T-C (> 15% of the samples, Table 3) and *PACRG* A-T-A-T (> 40% of the samples). The frequencies of the *PRKN* C-G-C-G-C-T-C, C-G-C-G-T-T-C, C-G-T-A-T-T-C, C-G-T-G-T-T-C, C-G-C-A-C-T-C, C-G-C-A-T-T-C, C-G-T-A-C-C-T, C-G-T-G-C-C-T, C-T-T-A-T-T-C, C-T-C-A-C-C-T and C-T-C-G-C-C-T; *PACRG* T-C-C-G, A-T-C-T and A-T-A-G haplotypes were distinctly different between the normal and hyperlipidemic groups. Meanwhile, the haplotypes of the *PRKN* C-G-C-G-C-T-C, C-G-C-A-C-T-C, C-G-T-A-C-C-T, C-T-T-A-T-T-C, C-G-C-A-T-T-C, C-G-T-G-T-T-C, C-T-C-G-C-C-T and C-G-T-A-T-T-C; *PACRG* A-T-A-G were related to an increased morbidity of hyperlipidaemia, whereas the haplotypes of the *PRKN* C-G-C-G-T-T-C, C-T-C-A-C-C-T and C-G-T-G-C-C-T; *PACRG* A-T-C-T and T-C-C-G were correlated with a protective effect (*P* < 0.05–0.001, respectively).

**Table 3:** Association between the haplotypes among 7 SNPs of the *PRKN* and 4 SNPs of the *PACRG* and hyperlipidaemia [*n* (frequency)].

Haplotype	Normal	Hyperlipidemia	*x*^2^	*P*	OR(95% CI)
*PRKN* C-G-C-A-C-T-C	33.75 (0.019)	43.17 (0.029)	4.088	0.043	1.621 (1.022–2.572)
*PRKN* C-G-C-A-T-T-C	39.47 (0.022)	74.07 (0.050)	22.911	1.75E-006	2.555 (1.719–3.797)
*PRKN* C-G-C-G-C–C-T	38.68 (0.021)	41.93 (0.028)	1.783	0.182	1.364 (0.871–2.135)
*PRKN* C-G-C-G-C-T-C	30.55 (0.017)	45.60 (0.031)	8.551	0.003468	1.982 (1.243–3.159)
*PRKN* C-G-C-G-T-T-C	136.68(0.075)	59.62(0.041)	15.298	9.30E-005	0.535 (0.390–0.735)
*PRKN* C-G-T-A-C–C-T	20.00 (0.011)	33.37 (0.023)	6.866	0.009	2.118 (1.206–3.719)
*PRKN* C-G-T-A-C-T-C	46.12 (0.025)	33.00 (0.022)	0.329	0.566	0.872 (0.552–1.379)
*PRKN* C-G-T-A-T-T-C	25.57 (0.014)	64.41 (0.044)	29.992	4.55E-008	3.431 (2.152–5.471)
*PRKN* C-G-T-G-C–C-T	211.41 (0.116)	47.92 (0.033)	75.539	3.44E-015	0.255 (0.184–0.354)
*PRKN* C-G-T-G-C-T-C	68.45(0.038)	61.64 (0.042)	0.822	0.365	1.178 (0.826–1.681)
*PRKN* C-G-T-G-T-T-C	284.29 (0.156)	294.34 (0.200)	17.205	3.41E-005	1.499 (1.237–1.815)
*PRKN* C-T-C-A-C–C-T	50.90 (0.028)	23.76 (0.016)	5.096	0.024	0.564 (0.343–0.928)
*PRKN* C-T-C-A-C-T-C	29.55 (0.016)	22.94 (0.016)	0.035	0.851	0.961 (0.552–1.672)
*PRKN* C-T-C-G-C–C-T	26.50 (0.015)	37.80 (0.026)	5.236	0.022	1.809 (1.090–3.002)
*PRKN* C-T-C-G-C-T-C	28.81 (0.016)	27.10 (0.018)	0.296	0.586	1.172 (0.687–1.998)
*PRKN* C-T-C-G-T-T-C	40.76 (0.022)	27.34 (0.019)	0.704	0.455	0.825 (0.503–1.352)
*PRKN* C-T-T-A-T-T-C	20.61 (0.011)	50.53 (0.034)	22.579	2.07E-006	3.301 (1.963–5.551)
*PRKN* C-T-T-G-C–C-T	26.68(0.018)	24.16 (0.013)	0.123	0.726	1.126 (0.644–1.970)
*PRKN* C-T-T-G-T-T-C	56.35 (0.031)	59.54 (0.040)	3.041	0.082	1.394 (0.958–2.028)
*PACRG* A-C-A-T	56.91 (0.031)	49.45 (0.034)	0.127	0.721	1.073 (0.728–1.581)
*PACRG* A-T-A-G	140.99 (0.077)	191.10 (0.130)	24.381	8.16E-007	1.775 (1.410–2.234)
*PACRG* A-T-A-T	792.83 (0.435)	654.85 (0.445)	0.203	0.653	1.033 (0.897–1.189)
*PACRG* A-T-C-T	388.97 (0.213)	248.25 (0.169)	11.041	0.001	0.741 (0.620–0.884)
*PACRG* T-C-C-G	238.67 (0.131)	150.14 (0.102)	6.868	0.008	0.748 (0.602–0.930)
*PACRG* T-C-C-T	115.33 (0.063)	113.41 (0.077)	2.277	0.131	1.230 (0.940–1.610)

The haplotypes of *PRKN* were composed in the order of rs10755582, rs9458363, rs2022991, rs9365344, rs1105056, rs4636000, and rs2155510 SNPs. The haplotypes of *PACRG* were composed in the order of rs2206256, rs11966842, rs11966948, and rs6904305 SNPs. *PRKN*, parkin RBR E3 ubiquitin protein ligase gene; *PACRG*, the parkin coregulated gene; Rare Hap (frequency < 1%) in both groups was dropped. *P* was obtained by unconditional logistic regression analysis.

### Gene–gene interaction-based association with hyperlipidaemia

The commonest gene–gene interaction haplotype was the *PRKN*–*PACRG* C-G-T-G-T-T-C-A-T-A-T (Table 4). The frequencies of the *PRKN*–*PACRG* C-G-C-G-T-T-C-A-T-A-T, C-G-T-G-T-T-C-A-T-A-T, C-G-T-G-C-T-C-A-T-A-T, C-G-T-G-C-T-C-A-T-C-T, C-G-C-G-T-T-C-A-T-C-T and C-G-T-G-T-T-C-A-T-C-T haplotypes were distinctly different between the normal and hyperlipidemic groups. The haplotypes of C-G-T-G-C-T-C-A-T-A-T and C-G-T-G-T-T-C-A-T-A-T were correlated with an increased morbidity of hyperlipidaemia, whereas the haplotypes of C-G-C-G-T-T-C-A-T-A-T, C-G-C-G-T-T-C-A-T-C-T, C-G-T-G-C-T-C-A-T-C-T and C-G-T-G-T-T-C-A-T-C-T were correlated with a protective role (*P* < 0.05–0.001).

**Table 4:** Association between the G × G interaction haplotypes among 11 SNPs of the *PRKN–PACRG* cluster and hyperlipidaemia [*n* (frequency)].

GxG interaction haplotypes	Normal	Hyperlipidemia	*x*^2^	*P*	OR (95% CI)
**A-B-C-D-E–F-G-H-I-J-K**					
C-G-C-A-C-T-C-A-T-C-T	25.74 (0.014)	24.41 (0.017)	0.233	0.630	1.180 (0.672–2.071)
C-G-C-G-C-T-C-A-T-C-T	19.82 (0.011)	15.34 (0.010)	0.047	0.828	0.928 (0.472–1.825)
C-G-C-G-T-T-C-A-T-A-T	67.69 (0.037)	20.20 (0.014)	9.029	0.003	0.464 (0.278–0.773)
C-G-C-G-T-T-C-A-T-C-T	36.51 (0.020)	14.54 (0.010)	5.422	0.020	0.492 (0.268–0.904)
C-G-T-A-C-T-C-A-T-A-T	33.06 (0.018)	19.04 (0.013)	1.433	0.231	0.706 (0.398–1.252)
C-G-T-G-C–C-T-A-T-A-T	28.76 (0.016)	36.03 (0.024)	3.149	0.076	1.566 (0.951–2.579)
C-G-T-G-C-T-C-A-T-A-T	28.47 (0.016)	42.85 (0.029)	7.593	0.006	1.959 (1.205–3.186)
C-G-T-G-C-T-C-A-T-C-T	38.25 (0.021)	16.17 (0.011)	5.103	0.024	0.511 (0.283–0.924)
C-G-T-G-T-T-C-A-T-A-T	46.35 (0.025)	235.67 (0.160)	330.594	0.00E + 000	16.927 (11.901–24.075)
C-G-T-G-T-T-C-A-T-C-T	157.12 (0.086)	24.74 (0.017)	54.278	1.99E-013	0.213 (0.137–0.332)
C-T-C-A-C–C-T-A-T-A-T	26.25 (0.014)	14.20 (0.010)	1.548	0.213	0.661 (0.343–1.275)
C-T-T-G-T-T-C-A-T-C-T	41.12 (0.023)	29.32 (0.020)	0.309	0.578	0.871 (0.536–1.416)

A, *PRKN* rs10755582 C > T; B, *PRKN* rs9458363 G > T; C, *PRKN* rs2022991 T > C; D, *PRKN* rs9365344 A > G; E, *PRKN* rs1105056 C > T; F, *PRKN* rs4636000 C > T; G, *PRKN* rs2155510 T > C; H, *PACRG* rs2206256 T > A; I, *PACRG* rs11966842 T > C; J, *PACRG* rs11966948 C > A; K, *PACRG* rs6904305 T > C; *PRKN*, parkin RBR E3 ubiquitin protein ligase gene; *PACRG*, the parkin coregulated gene; Rare Hap (frequency < 1%) in both groups was dropped. *P* was obtained by unconditional logistic regression analysis.

### Gene–gene/environment interaction effect on hyperlipidaemia

Generalized multifactor dimensionality reduction (GMDR) was used to evaluate the association between gene–gene/environment factor (including hypertension, diabetes, smoking, drinking and BMI) interactions and the risk of hyperlipidaemia after adjusting for covariates. A significant three-locus model (*P* < 0.001) involving the rs9458363-rs4636000-rs11966842 SNPs was noted (Table 5, indicating a potential SNP-SNP interaction among the three SNPs). In general, this model exhibited a testing accuracy of 65.51% and a cross-validation consistency (CVC) of 10 of 10. A significant three-locus model (*P* < 0.001) including rs4636000-rs11966842-BMI > 24 kg/m^2^ was observed (the testing accuracy of 72.67%, a CVC of 10 of 10). In addition, other significant models including the haplotype-haplotype (*PRKN* C-G-C-A-T-T-C, *PACRG* A-T-A-T and *PACRG* A-T-C-T) and haplotype-environment (*PRKN* C-G-T-G-T-T-C, *PACRG* A-T-A-T and BMI > 24 kg/m^2^), gene–gene (*PRKN–PACRG* C-G-T-G-C-T-C-A-T-A-T, C-G-T-G-C-T-C-A-T-C-T and C-G-T-G-T-T-C-A-T-C-T), and gene-environment (C-G-T-G-C-T-C-A-T-C-T, C-G-T-G-T-T-C-A-T-C-T and BMI > 24 kg/m^2^) interactions were also detected in this study.

**Table 5:** Different interactions among the SNPs, their haplotypes, genetic and environmental factors detected by GMDR analyses.

Locus no	Best combination	Training Bal.Acc	Testing Bal.Acc	Cross-validation consistency	*P*	*P
**SNP-SNP interaction**						
2	rs9458363-rs4636000	0.6383	0.6331	10/10	< 0.001	< 0.001
3	rs9458363-rs4636000-rs11966842	0.6881	0.6551	10/10	< 0.001	< 0.001
**SNP-environment interaction**						
2	rs9458363-BMI > 24 kg/m^2^	0.7036	0.7047	10/10	< 0.001	< 0.001
3	rs4636000-rs11966842-BMI > 24 kg/m^2^	0.7260	0.7267	10/10	< 0.001	< 0.001
**Haplotype-haplotype interaction**						
2	A-B	0.5909	0.5720	7/10	< 0.001	< 0.001
3	B-C-D	0.6202	0.6204	10/10	< 0.001	< 0.001
**Haplotype-environment interaction**						
2	D-BMI > 24 kg/m^2^	0.7234	0.7233	10/10	< 0.001	< 0.001
3	A-D-BMI > 24 kg/m^2^	0.7511	0.7513	10/10	< 0.001	< 0.001
**Gene–gene interaction**						
2	F-G	0.5792	0.5747	8/10	< 0.001	< 0.001
3	E–F-G	0.5887	0.5878	10/10	< 0.001	< 0.001
**Gene-environment interaction**						
2	E-BMI > 24 kg/m^2^	0.7170	0.7122	7/10	< 0.001	< 0.001
3	F-G-BMI > 24 kg/m^2^	0.7313	0.7285	10/10	< 0.001	< 0.001

A = *PRKN* C-G-T-G-T-T-C haplotypes; B = *PACRG* A-T-C-T haplotypes; C = *PRKN* C-G-C-A-T-T-C haplotypes; D = *PACRG* A-T-A-T haplotypes; E = *PRKN-PACRG* C-G-T-G-C-T-C-A-T-A-T; F = *PRKN-PACRG* C-G-T-G-C-T-C-A-T-C-T; G = *PRKN-PACRG* C-G-T-G-T-T-C-A-T-C-T. *P* was obtained by adjusting for height and weight. *P was obtained by 1,000 permutation tests. The haplotypes of *PRKN* were composed in the order of rs10755582, rs9458363, rs2022991, rs9365344, rs1105056, rs4636000, and rs2155510 SNPs. The haplotypes of *PACRG* were composed in the order of rs2206256, rs11966842, rs11966948, and rs6904305 SNPs.

**Figure 3:**
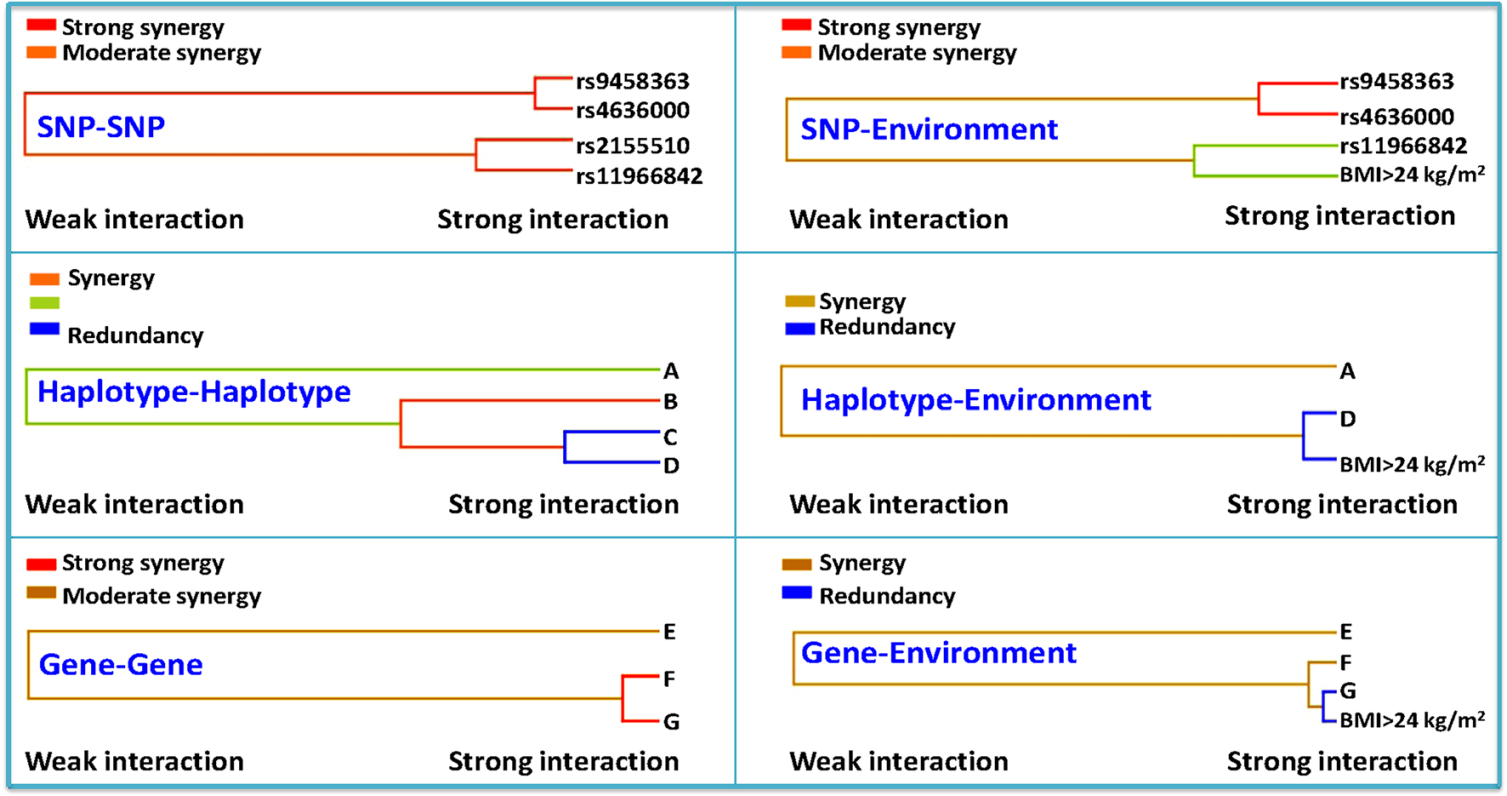
Different types of interaction dendrogram. The strongly interacting elements appear close together at the leaves of the of tree, and the weakly interacting elements appear distant from each other.

As shown in Fig. 3, the entropy-based interaction dendrogram established by MDR showed that the most powerful synergy existed for the *PRKN–PACRG* C-G-T-G-C-T-C-A-T-C-T and C-G-T-G-T-T-C-A-T-C-T (gene–gene) and rs9458363 and rs4636000 (SNP-SNP) interactions. The other interactions including rs11966842 and BMI > 24 kg/m^2^ (SNP-environment), *PRKN* C-G-C-A-T-T-C and *PACRG* A-T-A-T (haplotype-haplotype), *PACRG* A-T-A-T and BMI > 24 kg/m^2^ (haplotype-environment), and *PRKN–PACRG* C-G-T-G-T-T-C-A-T-C-T and BMI > 24 kg/m^2^ (gene-environment) were also detected. The 95% confidence intervals (CIs) and odds ratios (ORs) of the interactions determined via unconditional logistic regression analyses are shown in Table 6. The participants with the rs9458363 GT/TT and rs4636000 CT/CC genotypes exhibited a higher risk of hyperlipidaemia than those with the rs9458363 GG and rs4636000 TT genotypes (adjusted OR = 1.795, 95% CI = 1.352–2.383, *P* < 0.001). The subjects with the rs11966842 TT/CT genotype and a BMI > 24 kg/m^2^ presented an increased risk of hyperlipidaemia (adjusted OR = 1.12, 95% CI = 0.814–1.436, *P* = 0.024). The carriers of the *PRKN* C-G-C-A-T-T-C and *PACRG* A-T-A-T (adjusted OR = 1.191, 95% CI = 1.015–1.660, *P* < 0.001), *PACRG* A-T-A-T and BMI > 24 kg/m^2^ (adjusted OR = 1.327, 95% CI = 0.612–2.285, *P* < 0.001), and *PRKN–PACRG* C-G-T-G-T-T-C-A-T-C-T and BMI > 24 kg/m^2^ (adjusted OR = 1.066, 95% CI = 0.532–1.863, *P* < 0.001) increased the risk of hyperlipidaemia. But the carriers of the *PRKN–PACRG* C-G-T-G-C-T-C-A-T-C-T and C-G-T-G-T-T-C-A-T-C-T decreased the risk of hyperlipidaemia (adjusted OR = 0.172, 95% CI = 0.116–0.645, *P* < 0.001).

**Table 6:** Different types of interaction detected by logistic regression analyses.

Variable 1	Variable 2	OR (95% CI)	*P*
**SNP–SNP interactions**			
rs9458363	rs4636000		
GG	TT	1	–
GG	CT + CC	1.097 (0.844–1.426)	0.490
GT + TT	TT	1.105 (0.912–1.483)	0.368
GT + TT	CT + CC	1.795 (1.352–2.383)	5.27E-5
**SNP-environment interactions**			
rs11966842	BMI > 24 kg/m 2		
TT	No	1	–
TT	Yes	1.180 (0.816–1.429)	0.340
TT + CT	No	0.860 (0.762–1.136)	0.042
TT + CT	Yes	1.120 (0.814–1.436)	0.024
**Haplotype-haplotype interactions**			
*PRKN* C-G-C-A-T-T-C	*PACRG* A-T-A-T		
No-carriers	No-carriers	1	–
No-carriers	Carriers	1.070 (0.890–1.206)	0.091
Carriers	No-carriers	1.446 (0.978–1.775)	4.20E-4
Carriers	Carriers	1.191 (1.015–1.660)	5.16E-5
**Haplotype-environment interactions**			
*PACRG* A-T-A-T	BMI > 24 kg/m 2		
No-carriers	No	1	–
No-carriers	Yes	1.120 (0.805–1.756)	0.590
Carriers	No	1.761 (1.347–2.302)	3.53E-5
Carriers	Yes	1.327 (0.612–2.285)	2.89E-8
**Gene–gene interactions**			
C-G-T-G-C-T-C-A-T-C-T	C-G-T-G-T-T-C-A-T-C-T		
No-carriers	No-carriers	1	–
No-carriers	Carriers	0.374 (0.256–0.773)	0.031
Carriers	No-carriers	0.589 (0.331–1.047)	0.011
Carriers	Carriers	0.172 (0.116–0.645)	3.50E−4
**Gene-environment interactions**			
C-G-T-G-T-T-C-A-T-C-T	BMI > 24 kg/m^2^		
No-carriers	No	1	–
No-carriers	Yes	1.208 (0.661–2.353)	0.116
Carriers	No	0.758 (0.438–1.150)	0.030
Carriers	Yes	1.066 (0.532–1.863)	3.75E−5

*P* was obtained by adjusting for height and weight. The haplotypes of *PRKN* were composed in the order of rs10755582, rs9458363, rs2022991, rs9365344, rs1105056, rs4636000, and rs2155510 SNPs. The haplotypes of *PACRG* were composed in the order of rs2206256, rs11966842, rs11966948, and rs6904305 SNPs. Gender, cigarette smoking, blood pressure, alcohol consumption, blood glucose and age were adjusted for the statistical analysis.

## Discussion

The main findings of the current research included the following. (1) The frequencies of 11 *PRKN–PACRG* SNPs, their haplotypes and corresponding gene–gene inter-locus interactions in the Chinese Maonan nationality were revealed, which may provide a more complete complement to the 1,000 Genomes database. (2) Some new evidence about the potential *PRKN*–*PACRG* SNP-SNP/environment, haplotype-haplotype/environment, and gene–gene/environment interaction effects on blood lipid parameters was provided. (3) Several different effects of the *PRKN–PACRG* SNP-SNP/environment, haplotype-haplotype/environment and gene–gene/environment interactions on the risk of hyperlipidaemia were observed in our study populations.

The 2019 ESC/EAS guidelines for the management of dyslipidaemia indicate that the combined effects of LDL-C and other cholesterol-rich lipoproteins that accumulate in the subintima play a key role in the initiation of atherosclerosis, and the comprehensive reduction of lipid levels is more effective in reducing the risk of cardiovascular events than decreasing LDL-C levels alone, which is especially applicable to ACS patients with a high risk of cardiovascular events^18^. Many previous studies have also proven that hyperlipidaemia, a severe risk factor for CAD, may be due to the combined effects of various elements, such as age, sex, unhealthy lifestyles, genetic background, environmental factors and the interactions of these factors^16,19^.

The current research demonstrated the correlation between *PRKN* and *PACRG* SNPs and serum lipid parameters including LDL-C (rs11966842 and rs2155510), TC (rs1105056, rs10755582, rs2155510, rs11966842 and rs6904305), and TG (rs9365344 and rs11966948) in subjects with hyperlipidaemia and TG (rs10755582 and rs11966948) in the normal group. At the same time, we noted that the genotypic and allelic frequencies of the *PRKN* (rs10755582, rs9365344, rs1105056 and rs2155510) and *PACRG* (rs11966842, rs11966948 and rs6904305) SNPs were obviously different between the hyperlipidaemic and normal groups. When the relationship between the above SNPs and the onset of hyperlipidaemia was analysed, we found that the dominant models of the rs1105056, rs2155510, rs9365344 and rs6904305 SNPs increased the risk of hyperlipidaemia, whereas the dominant models of the rs10755582, rs11966842 and rs11966948 SNPs exhibited a protective effect. These results suggested that genetic factors were important risk factors for the prevalence of hyperlipidaemia. The reasons for these differences in the current study are not entirely clear. Differences in genetic factors and LD patterns may account for some of these differences.

The existence of multiple-locus LD indicates that nearby SNPs in adjacent genes are not statistically independent of each other genetically^10^. Strong LD was also found among the 11 SNPs (*r*^2^ = 0.01–0.64) examined in this research. We found that the most common haplotypes were *PRKN* C-G-T-G-T-T-C (> 15%) and *PACRG* A-T-A-T (> 40%). At the same time, the *PRKN* C-G-C-A-T-T-C haplotype was associated with an increased risk of hyperlipidaemia, whereas the interactions of the *PRKN–PACRG* C-G-T-G-C-T-C-A-T-C-T and C-G-T-G-T-T-C-A-T-C-T were correlated with a decreased risk of hyperlipidaemia. However, the assessment of SNP-environment interactions showed that the rs11966842 SNP and a BMI > 24 kg/m^2^ were associated with an increased risk of hyperlipidaemia in the participants. We also revealed that the occurrence of *PACRG* A-T-A-T and a BMI > 24 kg/m^2^ or *PRKN–PACRG* C-G-T-G-T-T-C-A-T-C-T and a BMI > 24 kg/m^2^ was related to an increased risk of hyperlipidaemia in the subjects. The above results indicated that correlation analysis based on haplotypes and gene–gene interactions could illuminate more changes in serum lipid levels compared to the analysis of a single SNP alone. Additionally, when the SNP/haplotype/gene-environment interactions were analyzed, we noted that a BMI > 24 kg/m^2^ could reverse the effects of the SNPs or haplotypes. A reasonable explanation for this finding may be that a genetic factor, combined with environmental and lifestyle factors, contributes to the development of hyperlipidaemia^16^.

The Maonan ethnic group is known in China for the unique marriage culture and eating habits of its members. The Maonan marriage culture is relatively conservative. Parents mainly arrange the marriages of their children. Maonans maintain the custom of intraethnic marriages, and intermarriage with other ethnic groups is rare. Therefore, the inherited features and genotypes of some lipid metabolism-associated genes in Maonans might differ from those in other populations. Rice is the staple food of Maonan people. They also eat corn, potatoes, wheat, sorghum, etc. The Maonan people especially prefer foods that are spicy, acidic and rich in salt as well as oil. The consumption of this type of diet, rich in long-chain highly saturated fat, might lead to high blood glucose levels, obesity, hyperlipidaemia, hypertension and atherosclerosis^20^. The main long-chain saturated fatty acids in the diet could produce harmful effects on blood lipid metabolism, impacting the levels of serum TG and TC in particular^21^.

Unhealthy lifestyle factors such as excessive drinking and cigarette smoking have been linked to hyperlipidaemia^22,23^. In the present study, we found that the percentage of the participants who smoked was greater in the hyperlipidaemic group than in the normal group. In recent years, the influence of smoking on hyperlipidaemia has attracted increasing attention. Several recent studies have indicated the existence of lower HDL-C and higher TC, LDL-C and TG levels in smokers compared to non-smokers^23^. Moderate drinking reduced the incidence of cardiovascular events, the potential mechanism may be related to increased HDL-C and ApoA1 levels^24^. However, the beneficial effect of drinking on HDL-C levels is negated by smoking. This may explain the difference in the serum lipid profiles between the two groups. Thus, the combined effects of various eating habits, lifestyle factors and environmental aspects may further alter the relationship between hereditary variations and serum lipid levels observed in the current study.

Several limitations of the current study were inevitable. First, the sample size was much smaller than those in previous large GWASes; therefore, further studies with a larger sample size are needed to confirm the findings. Second, although we examined the effects of 11 SNPs in the *PRKN–PACRG* cluster on lipid levels, numerous potential lipid-related SNPs were overlooked in the current study. Third, we did not determine the potential functional roles of the significant SNPs identified in the development of hyperlipidaemia; thus, the correlation of the findings needs to be validated by further in-depth studies with the incorporation of the genetic information of single-nucleotide mutations in the *PRKN*–*PACRG* cluster, their haplotypes, and G × G and G × E interactions via in vitro and in vivo functional studies to verify the effects of this variation at the molecular level, including the effects on transcription and translation.

In summary, there are potential correlations between *PRKN–PACRG* SNPs, environmental exposure and serum lipid parameters in the Maonan population. Furthermore, correlation analysis based on haplotypes and gene–gene interactions could improve the power of detecting the risk of dyslipidaemia compared with the analysis of any single SNP alone. The use of GMDR to analyse the interactions indicated that the different patterns of interaction identified between genetic and environmental factors result in different redundant or synergistic effects on the morbidity associated with hyperlipidaemia.

## Materials and methods

### Subjects

A total of 912 (405 males, 44.41%; 507 females, 55.59%) unrelated participants of normal lipid levels and 736 unrelated subjects (330 males, 44.84%; 406 females, 55.16%) of hyperlipidaemia were arbitrarily chosen based on our previously stratified randomized samples. All of the subjects were farmworkers who resided in Huanjiang Maonan Autonomous County, Guangxi Zhuang Autonomous Region of China. The age ranged from 22 to 88 years. There was not any difference in age distribution (55.83 ± 15.48 *vs*. 55.55 ± 14.33) and gender ratio between normal and hyperlipidemic groups. All participants were basically healthy and had no history of myocardial infarction, CAD, type 2 diabetes mellitus (T2DM) and ischemic stroke. They were not taking any medicines that could alter serum lipid levels. The selection criteria for Maonan individuals have been described in detail in our previous epidemiological studies^25,26^. In addition, all of the subjects were also confirmed by Y chromosome and mitochondrial diversity studies. All subjects had signed written informed consent. The research protocol was approved by the Ethics Committee of the First Affiliated Hospital, Guangxi Medical University (No. Lunshen-2014 KY-Guoji-001, Mar. 7, 2014).

### Epidemiological analysis

Universally standardized methods and protocols were used to conduct the epidemiological survey^27^. Detailed lifestyle and demographic characteristics were collected with a standard set of questionnaires. Alcohol consumption (0 (non-drinker), < 25 g/day and ≥ 25 g/day) and smoking status (0 (non-smoker), < 20 cigarettes/day and ≥ 20 cigarettes/day) were divided into three different subgroups. Waist circumference, BMI, height, blood pressure and weight were measured as previously described^28^.

### Biochemical assays

Fasting venous blood samples of 5 ml were collected from each subject. A portion of the sample (2 ml) was placed in a tube and used to measure serum lipid levels. The remaining sample of 3 ml was collected in a glass tube containing anticoagulants (14.70 g/L glucose, 13.20 g/L trisodium citrate, 4.80 g/L citric acid) and utilized to extract deoxyribonucleic acid (DNA). The methods for performing serum ApoA1, HDL-C, ApoB, TG, LDL-C and TC measurements were described in a previous study^29^. All determinations were conducted using an autoanalyzer (Type 7170A; Hitachi Ltd., Tokyo, Japan) in the Clinical Science Experiment Center of the First Affiliated Hospital, Guangxi Medical University^30,31^.

### SNP selection

Eleven SNPs in the *PRKN* and *PACRG* genes were selected according to the following criteria. (1) The *PRKN*–*PACRG* cluster, which is related to serum lipid levels, was chosen on the basis of previous GWASes. (2) Haploview (Broad Institute of MIT and Harvard, USA, version 4.2) was used to identify tagging SNPs, and the most recent version of the online 1,000 Genome Project Database was used to predict the functional SNPs that may be associated with lipid metabolism. (3) More complete information on the above SNPs was obtained from NCBI dbSNP Build 132 (https://www.ncbi.nlm.nih.gov/SNP/). (4) Regarding SNP selection, we also referred to a previous study by Ober et al.^11^; all selected SNPs have been associated with serum lipid parameters show a minor allele frequency (MAF) > 1%. (5) Eleven SNPs of *PRKN* (rs10755582, rs9458363, rs2022991, rs9365344, rs1105056, rs4636000 and rs2155510) and *PACRG* (rs2206256, rs11966842, rs11966948 and rs6904305) were selected via a block-based method. The strategy was implemented by noting the correlations of linkage disequilibrium (LD) between SNPs (*r*^2^ > 0.8).

### DNA amplification and genotyping

Genomic DNA was isolated from white blood cells in blood samples by phenol–chloroform method^29^. The extracted DNA samples were stored at 4 °C until experiment. Genotyping of the 11 SNPs was performed by the next-generation sequencing technology (NGS) at the Center for Human Genetics Research, Shanghai Genesky Bio-Tech Co. Ltd., China^32^. Detailed steps for multiplex PCR and high throughput sequencing and the primer sequences of the 11 SNPs are shown in supplementary materials.

### Diagnostic criteria

The values of serum ApoB (0.80–1.05 g/L), HDL-C (1.16–1.42 mmol/L), ApoA1 (1.20–1.60 g/L), TC (3.10–5.17 mmol/L), TG (0.56–1.70 mmol/L), the ApoA1/ApoB ratio (1.00–2.50) and LDL-C (2.70–3.10 mmol/L) were defined as normal at our Clinical Science Experiment Center. The subjects with TG > 1.70 mmol/L and/or TC > 5.17 mmol/L were defined as hyperlipidaemic^33^. The participants with the fasting plasma (blood) glucose value ≥ 7.0 mmol/L were defined as diabetes^34^. The diagnostic criteria of hypertension^35^, obesity, normal weight and overweight were referred to our previous study^36^.

### Statistical analyses

All data were evaluated using SPSS (version 22.0). The results are presented as the mean ± SD except for TG levels, which are presented as medians and interquartile ranges. Direct counting was used to determine allele frequency. The independent-samples *t* test was used to analyse the differences in general characteristics between the normal and hyperlipidaemic groups. The chi-square test was used to determine the genotype distribution between the two groups. HWE, pairwise LD (measured by *D*′), haplotype frequencies and gene–gene interactions were analysed using Haploview (Broad Institute of MIT and Harvard; version 4.2). The analysis of covariance (ANCOVA) was used to test the correlation between serum lipid parameters and genotypes, and *P* < 0.0045 (corresponding to *P* < 0.05 after adjusting for 11 independent tests by Bonferroni correction) was considered statistically significant. Unconditional logistic regression analysis was used to detect the associations between the haplotypes, genotypes and gene–gene interactions and the risk of hyperlipidaemia. Cigarette smoking, gender, blood pressure, BMI, alcohol consumption, blood glucose and age were adjusted for the statistical analysis. The best interaction combination among the SNP-SNP/environment, haplotype-haplotype/environment, and gene–gene/environment exposure interactions was screened with GMDR software (beta version 0.9, University of Virginia, Charlottesville, VA)^37^. GMDR reduces high-dimensional genetic data to a single dimension by exploring interaction models through cross-validation and using maximum likelihood estimates to calculate the score-based statistics of each participant. A CVC value close to 10 indicates that the module is good. The degree of CVC is an effective method for identifying the best model among all considered possibilities. A score between 0.50 (indicating that the model prediction results are no better than chance) and 1.00 (indicating perfect prediction) in the testing of balanced accuracy is an indicator that accurately predicts the extent of case–control status. Additionally, 1,000 permutations were performed to obtain permutated *P* values for these models. All SNPs, haplotypes and several environmental factors, such as hypertension, diabetes, smoking, drinking, and BMI, were included in the GMDR analysis. All of the above analyses were performed under the additive model adjusted for sex, age, and the study populations. A *P* value < 0.05 was considered to be statistically significant.

## Supplementary information

Supplementary information
